# Decoupling a novel *Trichormus variabilis*-*Synechocystis sp*. interaction to boost phycoremediation

**DOI:** 10.1038/s41598-019-38997-7

**Published:** 2019-02-21

**Authors:** Sepideh Abedi, Fatemeh Razi Astaraei, Barat Ghobadian, Omid Tavakoli, Hassan Jalili, H. Christopher Greenwell, Ian Cummins, Stephen Chivasa

**Affiliations:** 1 Department of Renewable Energies and Environmental Engineering, Faculty of New Sciences and Technologies, University of Tehran, Tehran, Iran; 2 Department of Biosystems Engineering, Tarbiat Modares University, Tehran, Iran; 3 School of Chemical Engineering, College of Engineering, University of Tehran, Tehran, Iran; 4 Department of Life Science Engineering, Faculty of New Sciences and Technologies, University of Tehran, Tehran, Iran; 5 Department of Earth Sciences, Durham University, Durham, DH1 3LE UK; 6 Department of Biosciences, Durham University, South Road, Durham, DH1 3LE UK

## Abstract

To conserve freshwater resources, domestic and industrial wastewater is recycled. Algal systems have emerged as an efficient, low-cost option for treatment (phycoremediation) of nutrient-rich wastewater and environmental protection. However, industrial wastewater may contain growth inhibitory compounds precluding algal use in phycoremediation. Therefore, extremophyte strains, which thrive in hostile environments, are sought-after. Here, we isolated such an alga - a strain of *Synechocystis sp*. we found to be capable of switching from commensal exploitation of the nitrogen-fixing *Trichormus variabilis*, for survival in nitrogen-deficient environments, to free-living growth in nitrate abundance. In nitrogen depletion, the cells are tethered to polysaccharide capsules of *T. variabilis* using nanotubular structures, presumably for nitrate acquisition. The composite culture failed to establish in industrial/domestic waste effluent. However, gradual exposure to increasing wastewater strength over time untethered *Synechocystis* cells and killed off *T. variabilis*. This switched the culture to a stress-acclimated monoculture of *Synechocystis sp*., which rapidly grew and flourished in wastewater, with ammonium and phosphate removal efficiencies of 99.4% and 97.5%, respectively. Therefore, this strain of *Synechocystis sp*. shows great promise for use in phycoremediation, with potential to rapidly generate biomass that can find use as a green feedstock for valuable bio-products in industrial applications.

## Introduction

Fresh water is a vital domestic and industrial resource, which also supports global food production systems and economic growth. Current trends in population growth, together with increased climate volatility and change, are putting pressure on fresh water supplies, making water a scarce resource. Withdrawals of freshwater in the last 50 years have tripled, with year-on-year demand increasing by 64 billion cubic meters^1^. Agriculture accounts for 70% of global water consumption^2^, with industrial and domestic sectors in close pursuit. However, considerable amounts (∼2.2 million m^3^ globally) of wastewater are generated annually from domestic, industrial, and agricultural processes^3^. To conserve water, recycling of wastewater is integral to water management systems and policies of many countries^4^.

Wastewater is rich in nutrients (C, N, P) and heavy metal ions (Pb, Zn, Cd, Cr, Cu and Ni)^5^ that could cause environmental degradation if released without removal. Microalgae thrive on such nutrients^6,7^ and so are appropriate biological systems for wastewater treatment. Algae are particularly desirable because they are photoautotrophs, with the capability to fix carbon, thereby mitigating the negative effects of greenhouse gas emissions^8^ and also rapidly generate biomass using nutrient-rich wastewater. Biomass can be used as a valuable source of energy, providing an opportunity to link wastewater treatment with microalgae cultivation and bio-energy production^9–16^.

Several studies demonstrated the utility of microalgae in wastewater treatment, for example, (i) several genera of green algae; *Chlorella*^17^, *Scenedesmus*^18,19^, *Haematococcus*, *Chloroccum*^20^*, Chlamydomonas*, *Micractinium*, *Oocystis* and *Tetraselmis*, and (ii) the blue-green algae *Oscillatoria* and *Phormidium*^21^. Combinations of microalgae with other organisms, such as with diatoms^22^ or a mixture of bacterial species^23^ have also shown effective nutrient removal from wastewater. In addition to utilising nitrate and phosphate^19,24^ for growth, the microalgal systems are also effective at removal of metals, such as Cd, Cu, Zn, Ni, Hg and Cr^25–28^, and metalloids, such as As, Sb, and Bi^29^. For example, *Chlorella vulgaris* efficiently removes both N and P^24^, and heavy metals^30^ from wastewater. Therefore, microalgal systems have emerged as a preferred wastewater treatment option due to their rapid growth and efficient nutrient and metal removal.

We have interest in screening for and developing dual-purpose algal strains, which can be used for wastewater treatment and generation of valuable products for industrial applications. In this study, we focused on *Trichormus variabilis* (syn. *Anabaena variabilis*)^31^, a freshwater filamentous Nostocoidae subfamily^32,33^ blue-green alga capable of fixing nitrogen. It can grow phototrophically, heteretrophically, or mixotrophically^34–36^ across a wide range of temperature^35,37^ and light^38^ conditions, making it a favorable microorganism to grow in variable conditions. In a nitrate-rich growth medium, filaments predominantly consist of vegetative photosynthetic cells, with akinetes appearing at the end of the exponential growth phase in response to nutrient limitation or stress cues^39,40^. However, in nitrate-depleted environments, 5–10% of the vegetative cells differentiate to form heterocysts^34^, whose main function is nitrogen fixation. Nitrogen fixation is catalysed by nitrogenase enzymes and generates molecular hydrogen as a by-product. Given the remarkable potential for bio-hydrogen generation^41,42^, incorporation of *T. variabilis* in microalgal wastewater treatment systems^43,44^ is commercially attractive.

A key incentive proposed for the use of microalgal systems in wastewater treatment is the potential for conversion of the biomass generated during treatment into different forms of bio-energy, including biogas^45^, biodiesel^20,22,46,47^ and bio-hydrogen^48^. This energy could be used to defray operational costs at waste treatment plants, or sold on local energy markets. The ability of *T. variabilis* to fix atmospheric nitrogen is of immense interest as one of the recognised shortfalls in using microalgae for biofuel production is the relative high cost of nitrogen versus the energy generated^46^. In this study, we wanted to explore the potential use of *T. variabilis* as a microalgal system suitable for coupling wastewater treatment to energy production. Although the study initially aimed to optimise cultivation of *T. variabilis* in wastewater, our results revealed an unexpected commensal relationship between *T. variabilis* and a strain of *Synechocystis sp*., which has remarkable stress resilience and an ability to grow and thrive in wastewater. The physical interaction of *T. variabilis* and *Synechocystis sp*. provides a new model system for studying cell-cell communications in biological systems.

## Results

### Acclimation for growth in wastewater

**Figure 1:**
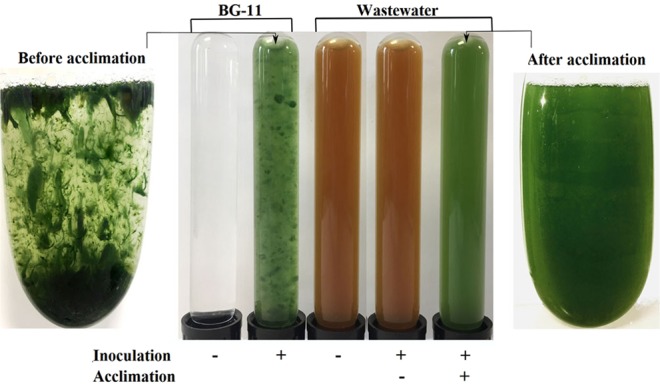
Acclimation of algal culture for growth in wastewater. Flasks with BG-11 medium or wastewater were mock-inoculated or inoculated with algal cells. Samples were photographed after a week of growth. Plus sign (+) denotes addition of the indicated inoculum; minus sign (−) denotes mock-inoculation.

Initial attempts to grow algal cells in the mixed domestic + industrial wastewater were futile. Algal cells failed to multiply and colonise the wastewater (Fig. 1). There was still no growth even after adjusting the wastewater pH from the pH 8–9 range down to the BG-11 growth medium pH 7.1. *T. variabilis* grows optimally in BG-11 medium. It was not clear whether failure to support algal growth was a result of lack of some essential nutrient(s) or the presence of growth inhibitory substances. We then made a 50% dilution of the wastewater with BG-11 growth medium as a way to supplement essential nutrients, which could be absent from the wastewater. Again there was no growth in this diluted wastewater (Fig. 1), suggesting that growth inhibition from components within the wastewater was the likely explanation of these results. Thus, we concluded that the wastewater contained growth inhibitory compounds affecting algal cell multiplication.

In order to survive in adverse environmental conditions, many prokaryotes and eukaryotes require an acclimation period during which the organism is first exposed to intermediate levels of the stress factor(s) to gradually change gene expression for growth under the extreme conditions^49,50^. We reasoned that exposure of *T. variabilis* cells to increasing concentrations of the wastewater could enable them to gradually acclimate and survive the “stress factors” present in the wastewater. Thus, BG-11 medium supplemented with 1% wastewater was inoculated with algal cells. The cells multiplied successfully and 7 days later, 1% wastewater culture was used to inoculate BG-11 medium spiked with 3% wastewater. Again there was successful growth of the cells in 3% wastewater. The sequential inoculation of progressively higher wastewater strength was repeated at 3 percentage point increases until we attained a stably growing algal culture in 50% wastewater over a 5 months period. Interestingly, attempts to increase wastewater strength by ≥5% at any point resulted in failure to grow, a strong indication that acclimation was vital for growth in increasing wastewater strength. Figure 1 shows acclimated algal cultures growing in wastewater.

But we noticed that algal cultures that had acclimated for growth in wastewater had a different appearance to original *T. variabilis* cultures growing in BG-11_0_ or BG-11 media. BG-11_0_ and BG-11 differ in that the latter contains the full complement of nutrients required for algal growth, while the former does not have nitrates. While control BG-11 cultures had thalloid or spherical clumps growth forms, acclimated cultures were homogenous cell suspensions (Fig. 1). When aeration is switched off from BG-11_0_ and BG-11 cultures, the algal sheets immediately settle to the bottom of the flask, while acclimated cells remain in suspension for several hours before finally sedimenting to the bottom of the flask. When the acclimated cells were inoculated back into BG-11 medium, they maintained the same culture properties, suggesting that acclimation had triggered long-lasting changes.

### *Synechocystis sp*. constitutes the acclimated cultures

**Figure 2:**
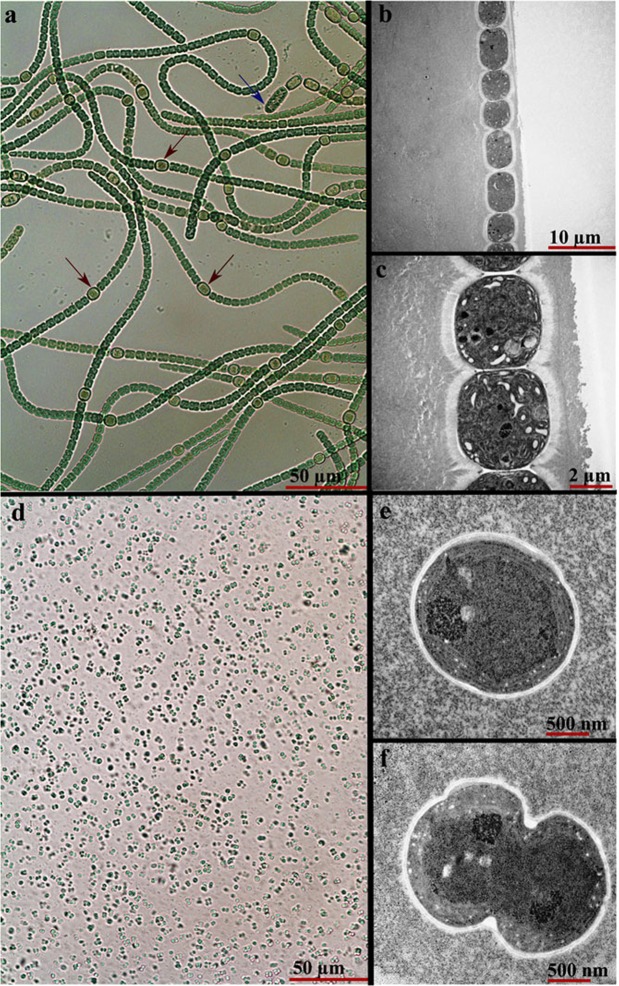
Microscopic images of cells from acclimated and non-acclimated algal cultures. (**a**) Filamentous cells not exposed to wastewater. Heterocysts and akinetes are indicated by red and blue arrows, respectively. (**b**) Vegetative cells in a filament from BG-11 cultures. (**c**) Higher magnification of vegetative cells in a filament. (**d**) Single cells in algal cultures acclimated for growth in wastewater. (**e**) Higher magnification of a single cell from the wastewater-acclimated culture. (**f**) Wastewater-acclimated single cell captured in the process of cell division.

To understand the basis for the change in appearance and physical properties of the acclimated cultures, we used microscopy to visualise the cells and make comparisons with the original *T. variabilis* cultures. Low power light microscopy of BG-11-grown cultures not exposed to wastewater revealed *T. variabilis* filaments with the typical vegetative cells, heterocysts and akinetes (Fig. 2a). In contrast, only single cells with no filaments were present in samples from acclimated cultures (Fig. 2d). This explains why the acclimated cultures were homogenous cell suspensions as opposed to the thalloid sheets in the non-acclimated cultures. The average vegetative cell diameter in filaments was 4.18 ± 0.54 μm (*n* = 10), while the diameter of acclimated single cells was 1.43 ± 0.53 μm (*n* = 10). Transmission electron microscopy (TEM) revealed detailed ultrastructural differences between vegetative cells of filaments from BG-11 cultures (Fig. 2b,c) and single cells of the acclimated cultures (Fig. 2e,f). Cells from acclimated cultures had pronounced folds of thylakoid membranes and many darkly-stained bodies. It is these single cells in the acclimated cultures that have resilience to as yet unidentified growth inhibitory compound(s) in wastewater, being able to rapidly multiply and attain very high biomass in a short space of time (~2.7 ± 0.1 mg.mL^−1^.day^−1^) through cell division, (Fig. 2f), instead of germination known to occur in akinete cells.

Transition of the filamentous *T. variabilis* culture to a rapidly growing, stress-resilient (SR) unicellular culture after acclimation raised questions. Was the original algal culture obtained from CCAP not axenic? Did it contain 2 distinct algal species – one filamentous and one unicellular? We decided to work on the hypothesis that, in addition to having *T. variabilis* filaments, a second unicellular alga might have constituted a very minor component of the original culture. This can easily be resolved via sequencing of genes used in bacterial taxonomy and identification.

Therefore, we isolated algal DNA and sequenced the 16S ribosomal RNA (rRNA) from filamentous non-acclimated samples and from acclimated unicellular samples. The sequence obtained from the filamentous culture was used as a query in BLAST searches against the entire NCBI nucleotide database and the top 5 hits, all with sequence identities above 99%, are displayed in Table 1. The top hit is *T. variabilis* and the others are either *T. variabilis* or *Anabaena sp*. This confirms that the filamentous alga is indeed *T. variabilis*. However, the sequence obtained from the unicellular algal cells had high identity with several *Synechocystis sp*. The top 5 candidates, with sequence identity at 100%, are various strains of *Synechocystis sp*. (Table 2). More importantly, aligning the *T. variabilis* and *Synechocystis sp*. 16S rRNA sequences led to a low sequence identity of 88.5%, confirming that the multicellular filaments and the single cell cultures are from two distinct algae. Taken together, these results show that the acclimated cell culture is a strain of *Synechocystis sp*., whose precise identity will require future whole genome RNA sequencing.

**Table 1:** BLAST search results of 16S rRNA gene sequence of filamentous algal cells.

Description	Query cover	E-value	Identity
*Trichormus variabilis* ES09-6 16S rRNA gene, partial sequence	100%	0.0	99%
*Trichormus variabilis* MACC-57 16S rRNA gene, partial sequence	99%	0.0	99%
*Anabaena sp*. SAG 28.79 16S rRNA gene and 16S-23S rRNA intergenic spacer, partial sequence	99%	0.0	99%
*Trichormus variabilis* NQAIF309 16S rRNA gene, partial sequence	99%	0.0	99%
*Trichormus variabilis* CCAP 1403/12 16S rRNA gene, partial sequence	99%	0.0	99%

**Table 2:** BLAST search results of 16S rRNA gene sequence of acclimated unicellular algal cells.

Description	Query cover	E-value	Identity
*Synechocystis sp*. MA01 16S rRNA gene, partial sequence	99%	0.0	100%
*Synechocystis sp*. IPPAS B-1465 chromosome, complete genome	99%	0.0	100%
*Synechocystis sp*. PCC 6803 sub-strain GT-G, complete genome	99%	0.0	100%
*Synechocystis sp*. KSU-WH-2 16S rRNA gene, partial sequence	99%	0.0	100%
*Synechocystis sp*. PCC 6714, complete genome	99%	0.0	100%

### *Synechocystis sp*. procures nitrogen via commensal interaction with *T. variabilis*

**Figure 3:**
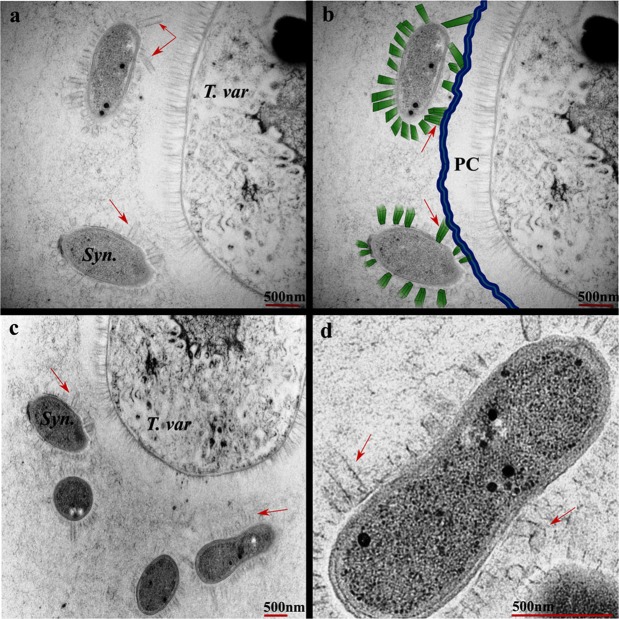
Interaction between cells of *T. v*ariabilis and *Synechocystis sp*. The micrographs show different sections with large *T. variabilis* and/or small *Synechocystis sp*. cells. (**a**,**b**) Show the same section, except that artificial colouring has been introduced in b to clarify different structural components. (**c**) Shows another section while (**d**) shows a magnified cell of *Synechocystis sp*. Nanotubular structures grow out of *Synechocystis sp*. cells and disappear into the polysaccharide capsule of *T. variabilis* cells. *T. var*, *T. variabilis*; *Syn*, *Synechocystis sp*.; PC, polysaccharide capsule. Arrows indicate the nanotubular structures. The samples analysed by TEM were harvested from exponential growth phase cultures grown in the N-deficient BG-11_0_ medium.

Our initial microscopic examination of the original cultures obtained from CCAP (Edinburgh, UK) and subsequent subcultures derived from these had revealed the presence of only filamentous *T. variabilis* cells with no unicellular *Synechocystis sp*. (Fig. 2). Thus, we reasoned that *Synechocystis sp*. must have been present as a very minor component, which acclimates and starts to increase in abundance when *T. variabilis* is killed off by toxic compounds in the wastewater. This could explain how a stress-resilient pure culture of *Synechocystis sp*. arose. Finding the elusive cells of *Synechocystis sp*. in the pre-acclimation *T. variabilis* culture would support this line of reasoning. Therefore, we prepared numerous additional sections of algal samples taken from N-deficient medium-grown non-acclimated culture (dominated by *T. variabilis* filaments) for TEM analysis. After examining several dozens of sections, we finally identified rare occurrences of cells of *Synechocystis sp*. within the BG-11_0_ cultures. They were invariably found in the immediate vicinity of *T. variabilis* vegetative cells with a very intriguing morphology. The cells had developed nanotubular outgrowths from the cell periphery, which tethered the cells to the polysaccharide capsule of neighbouring *T. variabilis* filaments (Fig. 3). This was very clear in instances where both the *Synechocystis sp*. and *T. variabilis* cells were in the same focal plane of the section.

**Figure 4:**
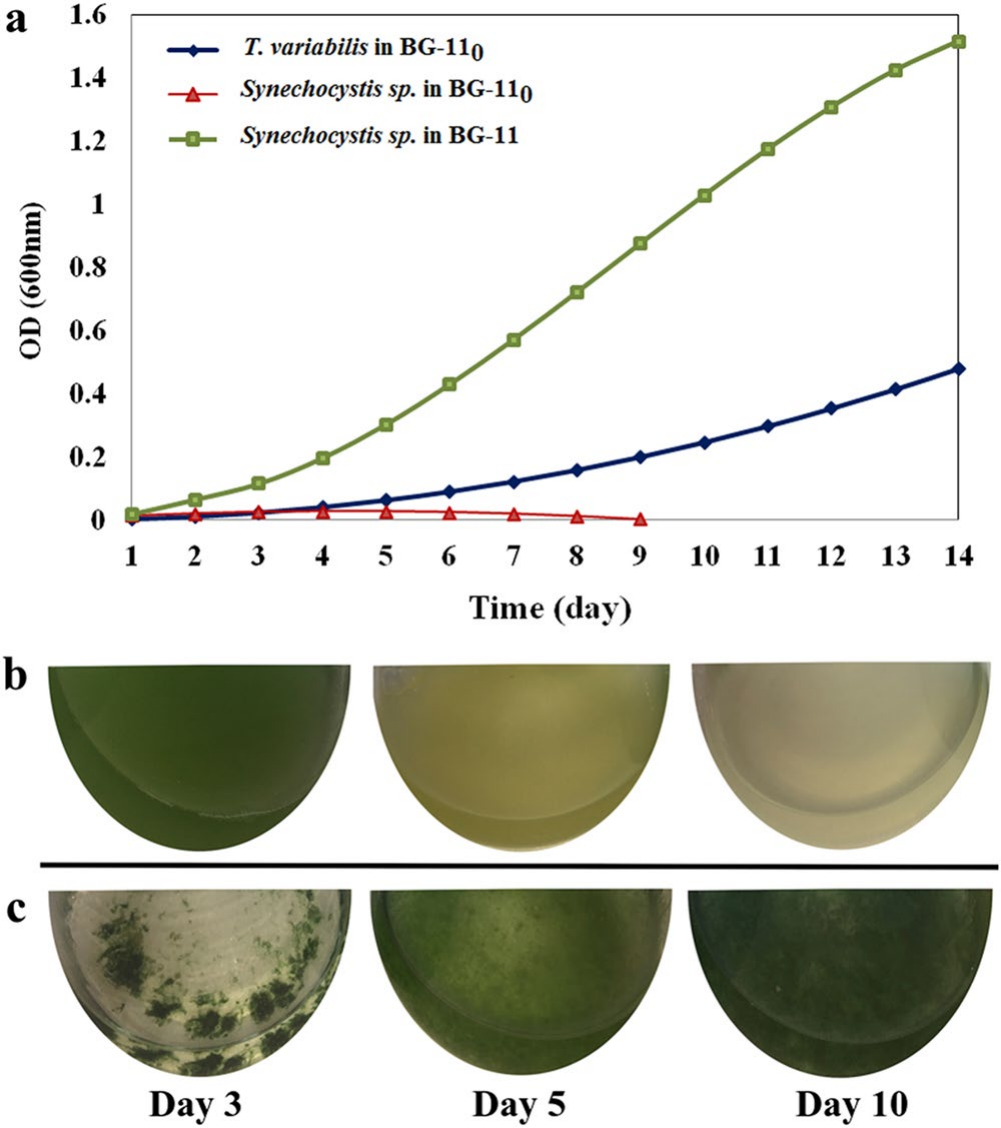
External source of nitrate is required for viability and growth of *Synechocystis sp*. (**a**) Growth curves of *Synechocystis sp*. in N-deficient BG-11_0_ and N-replete BG-11 growth media. *T. variabilis* growing in N-deficient BG-11_0_ medium was included as a positive control. (**b**) Gradual cell death of *Synechocystis sp*. grown in BG-11 after transfer to BG-11_0_. (**c**) *T. variabilis* culture grown in BG-11 and transferred to BG-11_0_ medium continues to grow.

*Trichormus variabilis* can switch between 2 modes of nitrate acquisition – uptake from the growth medium or fixing atmospheric nitrogen when growing in a medium bereft of nitrate. Acquisition of nitrogen is one likely reason why cells of *Synechocystis sp*. may attach to *T. variabilis* filaments in a commensal relationship, which does not harm the host. Therefore, we conducted experiments to investigate if *Synechocystis sp*. can grow in the absence of nitrate in the medium. *Synechocystis sp*. grew well in BG-11 medium (Fig. 4a), which has nitrates, but attempts to get growth in the nitrate-deficient BG-11_0_ medium were unsuccessful (Fig. 4a). When biomass grown in the optimal BG-11 medium (with nitrate) was transferred to the nitrogen-deficient BG-11_0_ medium, *Synechocystis sp*. cultures died after several days (Fig. 4b). *T. variabilis* cultures similarly grown in BG-11 and then transferred to BG-11_0_ not only survived, but continued to grow under the nitrogen deficiency conditions (Fig. 4c). Taken together, this demonstrates that *Synechocystis sp*. does not have the ability to fix atmospheric nitrogen and so has to assimilate nitrate from an external source. We speculate that the nanotubular physical connections seen between cells of *Synechocystis sp*. and *T. variabilis* filaments may effect material exchange (particularly nitrate) between the two organisms. In support of such a hypothesis are the observations that (i) in pure culture, *Synechocystis sp*. fails to grow when inoculated into N-deficient BG-11_0_ medium and (ii) *Synechocystis sp*. originally grown to high density in the N-containing BG-11 medium undergoes chlorosis and then dies if transferred to N-deficient BG-11_0_ medium (Fig. 4). Thus, *Synechocystis sp*. survives in N-deficient BG-11_0_ medium only when in association with *T. variabilis* filaments. However, its growth under such conditions is infinitesimally low, presumably as to be almost imperceptible.

### Potential for application in phycoremediation

We investigated the effectiveness of the stress-resilient *Synechocystis sp*. strain in nutrient removal from wastewater. Mixed domestic and industrial wastewater was inoculated with 1 g/500 mL (FW/V ratio) of culture. The cells grew very rapidly, giving dark green algal cultures. A week after inoculation, samples were withdrawn for analysis of ammonium, phosphates, and chemical oxygen demand. Comparison of the background ammonium and phosphate content of untreated effluent with post-treatment levels revealed 99.4 and 97.5% removal of these nutrients by acclimated cultures, respectively (Table 3). The effluent’s oxygen demand (COD) was also dramatically decreased by more than 40% (Table 3). Reduction of COD together with ammonium and phosphate are key targets for effluent treatment. Taken together, the increased growth of acclimated *Synechocystis sp*. (Fig. 4a) and its efficient nutrient removal (Table 3) demonstrate how this alga could be a very effective tool for effluent treatment.

**Table 3:** Wastewater parameters before and after treatment with acclimated *Synechocystis sp*.

Parameter	Untreated (mg/L)	Treated (mg/L)	Reduction (%)
NH_4_^+^	443	2.48 ± 0.01	99.44
PO_4_^−^	520	12.8 ± 0.5	97.53
NO_3_	410	24 ± 0.16	94.14
COD	1890	1115 ± 30.9	41.00

## Discussion

An impediment to use of algal systems for wastewater treatment is the presence of phytotoxic or growth-inhibiting compounds within certain streams of industrial and/or domestic waste. We found that our starting algal material was a composite culture of *T. variabilis* and a strain of *Synechocystis sp*., with the latter being a minor component. Neither of these algae survived when directly inoculated into a mixed domestic and industrial effluent. Our objective was to acclimate the culture for growth in the wastewater. Gradual exposure of the culture to minute quantities of the wastewater and increasing the quantities over time appeared to recapitulate the process of acclimation seen in nature. This phenomenon is wide-spread, having been reported in many organisms, including plants^49^ cyanobacteria^50^ and insects^51,52^. In all of these instances, the acclimation process is driven by changes in gene expression enabling the organism to survive under what previously was a threat to the organism.

Microscopy revealed that our starting composite algal culture switched from *T. variabilis* dominance to a culture exclusively consisting of *Synechocystis sp*. It is clear that *T. variabilis* was eliminated from the culture by toxic compounds within the wastewater. However, the nature of these phytotoxic and growth-inhibitory compound(s) in the mixed effluent remains unknown. From examination of the cultures, we noticed that *T. variabilis* cells were purged early during acclimation, at ≤3% wastewater strength. However, *Synechocystis sp*. remaining as a monoculture still continued to require step-wise small increments in wastewater strength, with large increases ≥5% not being tolerated. This indicates that *Synechocystis sp*. required gradual acclimation to grow in the wastewater. At this point, the biochemical basis for *Synechocystis sp*. acclimation is unclear and the subject of ongoing research. We found this organism to already have a fascinating innovation for survival in natural environments with limited nitrogen. We demonstrated that *Synechocystis sp*. cannot fix nitrogen and, in axenic culture under laboratory conditions, fails to grow or survive in the absence of an external nitrogen source. To solve this serious problem, the organism forged a strategic association with *T. variabilis* in nature, as we found in the CCAP culture obtained from the wild.

The vegetative cells of filamentous *T. variabilis* can differentiate into 2 cell types (akinetes and heterocysts) under specific environmental conditions^53^. Akinetes are survival cells usually appearing when there is stress, such as nutrient limitation, low light, or low temperature^40,54^. Heterocysts differentiate in response to depletion of external sources of nitrogen. Differentiation of heterocysts is accompanied by changes in cell morphology and expression of genes for the oxygen-sensitive nitrogenase enzymes responsible for nitrogen fixation. Only a few cells along each filament differentiate into heterocysts, which exchange nitrate for sugar with neighbouring photosynthetic vegetative cells along the chain. In the composite culture grown in nitrogen-depleted BG-11_0_ medium, we found cells of *Synechocystis sp*. nestling to *T. variabilis* vegetative cells. Since *Synechocystis* cells die when incubated in BG-11_0_ medium, their perpetual presence in the composite culture means they somehow derive their nitrate requirements from the nitrogen-fixing *T. variabilis*. Development of nanotubular structures on *Synechocystis* cells, hooking them to the polysaccharide capsule of *T. variabilis* filaments is a possible route for nitrate acquisition in what appears to us a commensal relationship. We speculate that the very low cell number of *Synechocystis sp*. in the composite culture ensures that nutrient withdrawals are not too high to prevent debilitating *T. variabilis* growth.

The nanotubular structures were not present in *Synechocystis* cells growing in the nitrogen-rich BG-11 medium or wastewater. Therefore, their development could be cued by the environmental nitrogen limitation. Morphological change entailing development of extracellular appendages hitching together neighbouring cells is not unprecedented in the *Synechocystis* genus. For example, *Synechocystis sp*. PCC 6803 was reported to develop such appendages in response to limited oxygen gas^55^. In fact this phenomenon was observed in other algae, such as the appendages connecting co-cultured cells of *Pelotomaculum thermopropionicum* and *Methanothermobacter thermoautotrophicus*, which are thought to effect cross-species material transfer^55,56^.

Changes in cell morphology in response to environmental cues are also prevalent in microorganisms beyond algae. For example, the wheat pathogen *Zymoseptoria tritici* can grow as filamentous hyphae or a yeast-like culture of unicellular micropycnidiospores^57^ as driven by external light cues. The micropycnidiospores are not dormant spores, but rather vegetative cells, which divide and grow in a yeast-like fashion. Darkness stimulates filamentous growth and light stimulates yeast-like growth^58^. While the change in morphotype of this fungus is cued by light, stress has previously been reported to serve as a stimulus triggering a change in bacterial morphology. For example, the loosely packed coccobacillus cells of the heterotrophic bacterium *Acidocella sp*. (strain GS19h) can switch to chains of coccidal lenticular cells or filaments when exposed to heavy metal stress^59^. In *T. variabilis* nitrogen depletion is a powerful cue for vegetative cell differentiation into heterocysts^53^.

The strain of *Synechocystis sp*. isolated here could be employed for commercial development of an algal wastewater treatment system. Rapid growth of acclimated *Synechocystis sp*. in wastewater led to efficient reduction of nutrients, such as ammonium and phosphate. The biomass can be used for fractionation of products for use in industrial applications. However, many aspects of algal growth and large-scale production require optimisation before developing this strain for industrial exploitation. Therefore, our study provides an avenue by which algae could be optimized for both wastewater treatment and as a resource for industrial feedstocks.

## Materials and Methods

### Cultivation of algae

The original culture of *Trichormus variabilis* CCAP 1403/4B was obtained from the CCAP collection (Edinburgh, UK) and cultivated in BG-11 (Blue-Green) culture medium according to Stainer *et al*.^60^. A litre of BG-11 growth medium contains; 1.5 g NaNO_3_, 40 mg K_2_HPO_4_, 75 mg MgSO_4_.7H_2_O, 36 mg CaCl_2_.2H_2_O, 6 mg citric acid, 6 mg ammonium ferric citrate green, 1 mg EDTANa_2_, 20 mg Na_2_CO_3_, 2.86 mg H_3_BO_3_, 1.81 mg MnCl_2_.4H_2_O, 220 µg ZnSO_4_.7H_2_O, 390 µg Na_2_MoO_4_.2H_2_O, 80 µg CuSO_4_.5H_2_O, and 50 µg Co(NO_3_)_2_.6H_2_O. For some experiments, cultures were also cultivated in BG-11_0_ medium^61^, which is N-depleted BG-11 medium with all metal nitrates replaced with CoCl_2_. Biomass was grown in sterilised glass tubes or flasks incubated with illumination at 65.5 μE.m^−2^ .s^−1^ and 25 °C. Stationary cultures were aerated by bubbling filter-sterilised air through a sterile glass tubing releasing air at the bottom of the culture vessels. Cultures in conical flasks were aerated by orbital shaking (LS-X, Adolf Kühner AG, Birsfelden, Switzerland) at 115 rpm.

### Transmission electron microscopy

For electron microscopy, cells were cryo-fixed by high-pressure freezing with a high pressure freezer EM ICE (Leica Microsystems Inc, Buffalo Grove, US). Super quick-freeze substitution was performed using 1% OsO_4_, 0.5% uranyl acetate and 5% H_2_O in acetone followed by embedding into epoxy resin^62^. Ultrathin sections (50 nm) were prepared using ultramicrotome UC6 (Leica Microsystems Inc, Buffalo Grove, US) contrasted for 10 min each in 1% uranyl acetate in ethanol followed by Reynolds lead citrate. Sections were viewed at 100 kV with a Hitachi H-7600 transmission electron microscope (Hitachi, Tokyo, Japan).

### Wastewater treatment and nutrient analysis

Mixed municipal plus industrial wastewater effluent (anaerobic digestate sludge liquor returns) were obtained from Bran Sands Waste Treatment facility of Northumbrian Water Ltd. (Middlesbrough, UK). At the waste processing plant, after anaerobic digestion the effluent is centrifuged, the solids are separated out and the liquor goes back into the plant for further treatment. Samples of this sludge liquor return were obtained and all endogenous organisms killed by autoclaving prior to use in experiments. The wastewater was sterilised by autoclaving prior to inoculation with *T. variabilis*. We acclimated the cultures for growth in wastewater via consecutive passaging through a serial dilution of increasing wastewater strength. Experiments were conducted using glass culture vessels incubated at 25 °C/65.5 μE.m^−2^ .s^−1^ and bubbling compressed air. Inoculums of 100 mg filamentous culture was added to 70 mL BG-11 containing - 1% (v/v) wastewater and allowed to grow for a week. An aliquot from this culture was used to inoculate a 3% wastewater-containing BG-11 medium. This process was repeated successively, with 3% wastewater increments each week until growth was stabilised at 50% wastewater. The acclimated culture was then used for subsequent experiments.

To evaluate the efficacy of algal cultures in wastewater treatment, triplicates of 500 mL wastewater were inoculated with 1 g of the acclimated algal culture. A week later, samples were withdrawn for determination of ammonium and phosphate content, and the chemical oxygen demand (COD) were analysed using LCK test kits according to the manufacturer’s instructions (Hach Lange, Manchester, UK).

### Sequencing 16S rRNA gene

The original algal culture obtained from CCAP was grown in BG-11_0_ medium and the biomass, which was dominated by *T. variabilis* filaments, harvested at the exponential growth stage for DNA extraction. An algal culture acclimated for growth in wastewater (as described above) was grown in BG-11 medium and harvested at the exponential growth phase for DNA extraction. This culture consisted entire of single cells with no filaments. DNA extraction and PCR analysis were performed as described previously^63^ using the primers Cya359F “GGGGAATYTTCCGCAATGGG” and “ACGGGCGGTGTGTAC”, where Y is any pyrimidine. The DNA samples were submitted to Sequetech (Mountain View, CA, USA) to perform the sequencing. To identify the species, the NCBI BLASTN 2.8.0+ program^64^ was used to search the entire NCBI database (https://blast.ncbi.nlm.nih.gov/Blast.cgi) using the nucleotide sequences as the query. For comparison of the sequence obtained from the filamentous algal culture to the sequence from the unicellular algal culture, we used the William Pearson’s LALIGN program, an online sequence alignment tool found on the EXPASY Bioinformatics Resource Portal (https://embnet.vital-it.ch/software/LALIGN_form.html), which utilizes an algorithm developed by Huang and Miller^65^.
